# Grading of Milk Supplies

**Published:** 1916-12

**Authors:** S. W. Bateson

**Affiliations:** Acting Chief, Bureau of Food and Drugs, Buffalo


### Grading of Milk Supplies.

S. W. BATESON, Acting Chief,

Bureau of Food and Drugs, Buffalo.

To distribute a milk supply which constantly conforms to the requirements of the New York State Sanitary Code for what is termed "Grade A Pasteurized" milk, presupposes that the dairy farms, on which milk is produced, conform to score requirements of not less than 25 out of a possible 40 points for equipment and 43 points out of a possible 60 for methods; that the cows shall be healthy, as disclosed by an annual physical examination by an approved veterinarian; that the milk shall not, at any time previous to pasteurization, contain more than 200,000 bacteria per cubic centimeter, and not to exceed 30,000 bacteria per cubic centimeter at any time after pasteurization and previous to its delivery to the consumer.

Two years of rigid investigation and application of legal procedures, have demonstrated the impracticability of obtaining strict and constant compliance with these technical requirements, and for very pertinent reasons.

During the winter months, when farmers have time to attend to their dairy interests, they may and generally do, maintain very good sanitary conditions of barns, milk houses, yards, cattle, milk utensils, and, being supplied with a plentiful supply of chilled water for refrigerating milk supplies, are able to conform, from this standpoint, with score requirements, but with the approach of the season for general farming, there is a rush of work which demands their time and attention; dairy work is neglected, barns are not regularly cleaned, cows become dirty and are not cleaned, manure, if removed from the barn, is deposited at the most accessible point, generally in the barnyard, in close proximity to the barn; farm hands come from the fields, and, in many instances, with unwashed hands, milk the cows into pails which have not received the proper cleansing since their last use; the milking of cows is the last operation of a hard day's work, and the milkers are often too tired to prolong the work, hence the necessary process of effective cooling is neglected, the milk being deposited in city dealers' cans and perhaps set in a tank of water to slowly cool as best it may. The morning milking is just as hurriedly performed and the night's and morning's milk supplies rushed to the station for shipment to the city.

This is a picture of the general conditions, milk production being considered of secondary importance to other farm operations, and especially with labor hire at a premium.

Thus, the dairy farm score is lowered, the bacterial content of the milk increased, and the milk supply is no longer of a character to pass into the ''Grade A Pasteurized'' class.

An annual physical examination does not guarantee the healthfulness of the cattle beyond the time of examination, and there are many evidences of disease and injury discovered in the milk supply.

These conditions prevail throughout the spring, summer and autumn and are beyond the control of the inspector, who can only make very infrequent inspections, and leaves with the farmer a score card with recommendations for correcting deficiencies.

From the standpoint of structural conditions, the dairy farm situation has vastly improved during the past 4 or 5 years, and is steadily improving, but the attitude of farmers as to methods employed offsets the benefits which might be derived from the improved plants.

This is written not merely to censure the dairyman for his apparent indifference, for, in the majority of instances, he is at the mercy of conditions which he cannot control, but rather to prove my contention that, under general dairying conditions, as they exist in Western New York, it is impossible to obtain milk supplies, in any considerable quantity, which conform to ''Grade A Pasteurized'' milk requirements, and to obtain a product of ''Grade A Raw'' quality, is even a more difficult task.

It has, therefore, devolved upon the department of health to order a regrading of milk supplies to more nearly meet actual conditions.

I have made mention of the fact that an annual physical examination of cattle does not guarantee freedom from disease.

Any raw milk may be infected with pus bacteria, from a wounded or inflamed udder or teat or from suppurating wounds on other parts of the body; nor can such milk be guaranteed free from infections of tuberculosis, scarlet fever, diphtheria or any of the multitude of infectious or contagious diseases to which man is heir.

Therefore, it is evident that some protection must be afforded to consumers of the lacteal fluid, and that protection is procured by perfect pasteurization followed by rapid cooling to a temperature below 50° F., and preferably in the final package to guard against possible recontamination.

The foregoing statement implies that the branding of our general milk supplies as of a grade above ''Grade C'' may be a misrepresentation, not necessarily intentionally; but nevertheless, a fact, which, I believe, calls for a revision of the code as it relates to the grading of milk supplies.

The interest of health officials is to secure to the public, a product as clean and safe as it can be produced, and without the misleading features of a grading system.

Knowing the facts about milk production, it is not difficult to conceive of the evil possibilities of consuming any raw supply, for the discovery is made that not even "Certified" milk is of known absolute purity at all times.

A milk supply, containing not to exceed 500,000 bacteria per cubic centimeter, free from pathogenic bacteria, may be characterized as a safe milk, but who knows whether it is free from infectious elements, for it is impossible to test every particle of milk daily; hence, again, the necessity for emphasis on perfect pasteurization; but no pasteurized milk for sale to the public should contain, at any time, more than 100,000 bacteria per cubic centimeter.

With a modification of the code to provide for a supply of milk not to exceed 500,000 bacteria per cubic centimeter before pasteurization, and not to exceed 100,000 bacteria per cubic centimeter, at any time prior to delivery to consumer, the municipal health department could demand a fairly strict observance, interdicting the supplies of any dairyman found to be persistently derelict, and penalizing or prohibiting milk dealers who fail to obtain specified results of pasteurization.

In any event, the need is for perfect pasteurization of all milk supplies, which should not be difficult, as, already, approximately ninety per cent of the supply of our city is being pasteurized and that without legal requirements.

---

**Alkaloidal Affinities of Hydrous Aluminum Silicate.** John Uri Lloyd, Ec. Med. Jour., Sept. Adsorption of various alkaloids, with consequent removal of their toxic properties, is demonstrated both by animal experiments and quantitation after treatment. From Fuller's earth Lloyd has prepared a reagent analysing approximately 17.41% water, 55.30% Si.02, 9.82% Al 223, 14.18% Fe 203, 1.58% Ca 0. Various analyses of Fuller's earth, kaolin and other clays are given and although, barring impurities, the analyses do not vary greatly, the adsorbent properties of many are almost nil. The reagent, i.e. the anhydrous aluminum silicate is not affecting by drying or treatment with RC1 HNO3 or aqua regia but the silicate has its water of crystalization driven off at a red heat. The reagent is employed for the extraction of alkaloids from crude drugs but, while of some value in the treatment of poisoning, is not claimed to protect against large doses.

